# Supplementary material for: Establishing biomarkers and clinical endpoints in myotonic dystrophy type 1 (END-DM1): Protocol of an international natural history study
Source: PLoS One. 2025 Dec 11;20(12):e0331163. doi: 10.1371/journal.pone.0331163 (PMC12697934; doi:10.1371/journal.pone.0331163)
Supplement: S3 Protocol — (DOC) [file pone.0331163.s003.doc]

**Protocol title: DMCRN Protocol:** **Establishing Biomarkers and Clinical Endpoints in Myotonic Dystrophy Type 1 (END-DM1)**

**Investigators of the Myotonic Dystrophy Clinical Research Network:**

Charles Thornton, MD, University of Rochester

Erika Simpson, MD, Houston Methodist

Jeffrey Statland, MD, Kansas University Medical Center

John Day, MD PhD, Stanford University

David Arnold, MD, Ohio State University

Ami Mankodi, MD, National Institutes of Health

Nicholas Johnson, MD, University of Utah

SH Subramony, MD, University of Florida

Laurie Gutmann, MD, University of Iowa

**Principal Investigator:**

Charles Thornton, MD

601 Elmwood Avenue, Box 645

Rochester, NY 14642

1. **PURPOSE OF THE STUDY AND BACKGROUND**
   1. **Purpose of the study**
      1. Characterize baseline status and disease progression over two years in 500 DM1 patients with myotonic dystrophy type 1 (DM1). Selected functional tests and patient-reported outcomes will be assessed for ability to quantify disease burden, detect disease progression, and predict changes.
      2. Optimize sample collection and analysis procedures for use of muscle RNA alternative splice events as biomarkers of DM1 severity. This analysis will be carried out on a subset of 60 participants who will undergo two muscle biopsies 3 months apart.
      3. Identify genetic modifiers of DM1 severity by performing a genome-wide association (GWA) study.
   2. **Background**

Mechanistic studies of DM1 have revealed a novel RNA-mediated disease process. The field has reached consensus that RNA toxicity is the core mechanism for DM1 pathogenesis. Good molecular targets have been identified, and recently there has been rapid progress in developing targeted therapies. Studies have shown that RNA toxicity is reversible in mouse models, encouraging us to believe that targeted treatments may have transformative effects on DM1 patients. However, recent meetings of stakeholders, including representatives from FDA, industry, academia, and advocacy, have indicated that three obstacles stand in the way of capitalizing on this opportunity: (1) insufficient data on natural history; (2) lack of reliable biomarkers; and (3) incomplete characterization and limited biological understanding of the phenotypic heterogeneity of DM1.

Building on previous work of the Myotonic Dystrophy Clinical Research Network (DMCRN), the present study seeks to overcome these roadblocks. Previously we used long study visits and extensive data collection to assess small cohorts of patients (80 subjects in the first study, 113 in the next). In the current study we are transitioning to focused data collection on a larger cohort. This will expand the scope of natural history data and help us develop strategies for managing patient heterogeneity going forward. For example, we hope to identify clinical measures or patient characteristics that predict future disease progression. In addition, we have made substantial progress in developing biomarkers of DM1 severity and therapeutic response, based on RNA alternative splicing in muscle tissue. We carried out large discovery studies to comprehensively identify DM1-associated splicing defects, then we developed high-throughput assays, and now we are examining test-retest reliability and associations with neurologic impairment. Although in studies to date the splicing biomarkers exhibited good reliability, we observed unexpected variance in some patients. Accordingly, the current study will examine strategies to improve the reliability by making further refinements in our sample collection and analysis procedures. Finally, we are studying the heterogeneity of DM1, and proposing that severity of disease is modified by common genetic variants. We will examine this possibility by performing GWA analysis in the cohort of 500 patients. If successful, this effort may lead to the identification of new stratification markers and therapeutic targets.

1. **STUDY DESIGN**
   1. **Overview**
      1. Investigators and sites

Tetsuo Ashizawa, Houston Methodist Medical Center

John Day, Stanford University

Nicholas Johnson, University of Utah (co-principal investigator)

John Kissel, Ohio State University

Ami Mankodi, National Institutes of Health

Jeff Statland, Kansas University Medical Center

S.H. Subramony, University of Florida

Charles Thornton, University of Rochester (co-principal investigator)

Laurie Gutmann, University of Iowa

- 1. **Rationale for Study Design**

Approximately 500 adult patients (18 to 70 years old, inclusive) with DM1 will be enrolled at 9 centers (up to 56 patients will be recruited at each site). No treatment will be administered as part of this study. Patients will receive standard of care as determined by their treating physician. Study visits occur at baseline/0 months, 12 months, and 24 months. Few restrictions are placed on participation in the study because we aim to capture the full spectrum of disease severity. Studies of splicing biomarkers in muscle biopsy samples will be conducted on a subset of 60 participants. These patients will have an additional study visit at 3 months.

1. **CHARACTERISTICS OF THE RESEARCH POPULATION**
   1. **Subject Characteristics**
2. **Number of Subjects:** This study will enroll 500 DM1 participants across 9 sites and 56 subjects will enroll locally at the University of Rochester.
3. **Gender and Age of Subjects:** DM1 has a prevalence rate of approximately 1 per 7,500. There are no expected gender differences. Both men and women will be selected for this study.

Children with DM1 are not included in this project because the pathophysiological basis of congenital and childhood DM1 appears to be mechanistically distinct.

1. **Racial and Ethnic Origin:** Prevalence rates of DM1 in major ethnic populations are not documented within the U.S. However, DM-1 prevalence rates in Africa are much lower than those in Europe and the US^1^. The demographics of myotonic dystrophy in Monroe County are not known. However, the latest demographics of Monroe County (White 77.0%, Black 16.2%, Hispanic 8.5%, Asian 3.7%, Native American 0.1%) indicate that our area contains minority populations similar to the national distribution (https://www.census.gov/quickfacts/fact/table/monroecountynewyork/PST045216). We have based our recruitment projections to reflect the demographics of Monroe County which closely parallel those of the general U.S. population.

The relative gender and minority balance in the Registry will enable us to more quickly and easily recruit patients to the University of Rochester.

- 1. **Inclusion and Exclusion Criteria**

1. **Inclusion Criteria:**

***Inclusion criteria***:

- Age 18 to 70 (inclusive)
- Competent to provide informed consent
- Clinical diagnosis of DM1 based on research criteria^1^ or positive genetic test
- *Comment:* The clinical research criteria require myotonia, muscle weakness in a characteristic distribution, and history of similar findings in a first degree relative. Genetic testing confirmed the diagnosis of DM1 in > 99% of individuals who satisfied these criteria.^2^

***Inclusion criteria for participants in the muscle biopsy sub-study:***

- Of the 60 patients undergoing biceps muscle biopsy, at least half will have at least moderate weakness of elbow flexion, defined as MRC score ≤ 4+. This is in order to obtain a muscle tissue sample in a person more severely affected with myotonic dystrophy. Approximately 10 patients at the University of Rochester will undergo the muscle biopsy.

1. **Exclusion Criteria:**

***Exclusion criteria***:

- Symptomatic renal or liver disease, uncontrolled diabetes or thyroid disorder, or active malignancy other than skin cancer.
- Current alcohol or substance abuse
- Concurrent enrollment in clinical trial for DM1, or participation in trial within 6 months of entry.
- Concurrent pregnancy or planned pregnancy during the course of the study.
- Concurrent medical condition that would, in the opinion of the investigator or clinical evaluator, compromise performance on study measures.
- Note: non-ambulatory participants are not excluded, but are limited to <15% of enrollment.

***Exclusion criteria for 60 participants in the muscle biopsy sub-study:***

- Known CTG repeat expansion size less than 100 repeats, unless there are clear cut signs of limb weakness and muscle wasting. This is in order to obtain a muscle tissue sample in a person more severely affected with myotonic dystrophy.
- Use of anticoagulant such as warfarin or a direct oral anticoagulant (e.g. dabigatran) due to the increased risk of bleeding.
- Use of aspirin or non-steroidal anti-inflammatory agents should be discontinued 3 days prior to the biopsy procedure, if possible.
- Platelet count <50,000 (if known) due to the increased risk of bleeding.
- History of a bleeding disorder due to the increased risk of bleeding.
- Advanced wasting of tibialis anterior (TA) muscle that precludes needle muscle biopsy in order to ensure that a sample taken would be of muscle and not just fat and fascia.
- Previous muscle biopsy of either TA in order to provide muscle tissue samples of non-biopsied (virgin) muscles.
  1. **Discussion of Subject Population**

Experienced investigators will be responsible for recruitment and follow-up of 500 subjects for this study. Participants will be men and women 18 to 70 with a positive genetic test for myotonic dystrophy type 1 or a clinical diagnosis of myotonic dystrophy type 1 (The clinical research criteria require myotonia, muscle weakness in a characteristic distribution, and history of similar findings in a first degree relative). Complete inclusion and exclusion eligibility criteria are listed above. To be eligible for enrollment into this study, subjects must meet all of the following eligibility criteria.

.

1. **SUBJECT IDENTIFICATION, RECRUITMENT AND CONSENT**
   1. **Method Of Subject Identification And Recruitment**

Volunteers will be recruited using local and nationwide recruitment tools. These individuals will be evaluated at one of the sites in the DMCRN. Recruitment media will include: recruitment websites and newsletters (e.g., Strong Health’s Clinical Trials website, the Muscular Dystrophy Association, and the Myotonic Dystrophy Foundation (MDF), clinicaltrials.gov, and direct mailings through the *National Registry of Myotonic Dystrophy and Facioscapulohumeral Dystrophy Patients and Family Members* (RSRB #12163).

## *4.1.a. Pre-entry Screening*

The study coordinator will make a provisional determination of ambulatory status and absence of exclusion criteria. Given that some patients who do not reside close to the study site will be recruited, the Investigator may elect to review a prospective participant’s medical records prior to Visit 1 in the study. . If this is necessary, subjects will be mailed a medical record release form for them to sign. Once the coordinator receives the signed release form they will request the medical records to review. Prospective participants who appear eligible will be scheduled for a Screening/Baseline Visit.

- 1. **Process of Consent**

The consent form will be mailed to the subject prior to the CRC visit so they have time to read the consent form and share the information with their family members. Informed consent will be obtained by the principal investigator or study coordinator after details of the study and its requirements have been presented, and there has been an opportunity for the participant to ask questions. Consent will be obtained only after complete information has been provided to the participant about the purpose of the study, the procedures, risks, benefits, contact persons, compensation, and care for injury, and voluntary participation. The participant and study personnel obtaining consent will sign the IRB approved consent form. The participant will receive a copy of the consent form after it has been signed. The original will be retained in the files of the investigator.

1. **METHODS AND STUDY PROCEDURES**
   1. Overview of Study Visits **(table 1)**

Obtain consent (30 minutes)

Medical history (15 minutes)

Patient reported outcomes (60 minutes)

Functional measures (90 minutes)

Electrocardiogram (10 minutes)

Blood draw (10 minutes)

| **Table 1. Schedule of Events^A^** | | | | |
| --- | --- | --- | --- | --- |
|  | Baseline/0 month | 3 months^B^ | 12 months | 24 months |
| **Medical History** | X |  | X | X |
| **Urine pregnancy test^C^** | X | X | X | X |
| **Vital signs** | X | X | X | X |
| **Physical exam** | X | X | X | X |
| **Myotonic dystrophy health index** | X | X | X | X |
| **Eat-10 questionnaire** | X | X | X | X |
| **Cogstate battery** | X |  | X | X |
| **Domain delta questionnaire** |  | X | X | X |
| **Quantitative myometry** | X | X | X | X |
| **Manual muscle testing** | X | X | X | X |
| **Iowa oral performance instrument** | X | X | X | X |
| **Forced vital capacity** | X | X | X | X |
| **Timed supine to sit** | X | X | X | X |
| **Timed up and go** | X | X | X | X |
| **10 Meter Walk/Run** | X | X | X | X |
| **4-stair climb** | X | X | X | X |
| **Step test** | X | X | X | X |
| **Grip strength** | X | X | X | X |
| **9-hole peg test** | X | X | X | X |
| **Picking up coins** | X | X | X | X |
| **Hand opening time and hand held myotonia instrument** | X | X | X | X |
| **Blood draw** | X |  | X | X |
| **Muscle biopsy (60 participants only)** | X | X |  |  |
| **Electrocardiogram** | X |  | X | X |

1. The order of procedures for the Clinical Evaluator is provided in Appendix 1.
2. The 3 month visit only pertains to the 60 participants in the muscle biopsy sub-study.
3. For women of childbearing potential

**Clinical Assessments**

- 1. Medical History

*Medical History.* A structured medical history will be used to capture phenotypes for the GWA study. Particular attention will be paid to age of onset. This information will be recorded on a specific CRF designed to ask questions in an open-ended fashion, followed by specific questions centered around the onset of myotonia, muscle weakness, cataracts, and cognitive or sleep symptoms. Common comorbidities will be documented. Specifically, sleep apnea, use of non-invasive ventilatory support or CPAP, presence of a pacemaker or ICD, history of bowel obstruction or pseudo-obstruction, and presence of thyroid disease or diabetes mellitus will be recorded.

*Urine Pregnancy Test.* Women of childbearing potential will have a urine pregnancy test. If the test is positive at the baseline visit, they will not be enrolled in the study. If the test is positive at the follow-up visits, the visit will be stopped and the participant will be asked to return at the next appropriate study visit that falls 6 months or later after delivery.

*Vital Signs.* Height, weight, blood pressure, pulse, respiratory rate, and temperature will be obtained at each study visit.

*Physical Exam.* An abbreviated physical exam will be performed by the study physician at each study visit.

- 1. Functional Measures
     1. *Upper Extremity Functional Outcome Measures*

*Grip Strength*. Participants will complete two trials of maximum hand grip using a JAMAR+ digital dynamometer on right and left hands. The maximum value will be recorded for each hand.

*Video Hand Opening Time and Handheld myotonia instrument.* To eliminate “warm-up” of myotonia the participants will rest the right hand for five minutes. The participant will squeeze the hand for three seconds then open the hand as quickly as possible. A video recording of the procedure will be obtained showing the forearm and hand but not the face. Recordings will later be assessed by a blinded reviewer to determine the hand opening time. Hand myotonia will also be assessed using a handheld force transducer, to determine the time required for isometric grip or pinch force to relax from 90% to 5% of peak force.

*Picking Up Coins*. Individuals will pick up 6 coins (one at a time) and hold them in their hand. The time to perform the task will be recorded.

*9-Hole Peg Test.* This test will be performed in accordance with standardized instructions. Two trials will be collected using the participant’s dominant hand. The best time and average will be recorded.

- - 1. *Measures of gait and mobility*

*10 meter walk/run*. From a standing start the participant will be asked to go 10 meters as quickly as possible, whether by walking or running. Orthotics and ankle braces are allowed. The time in seconds to complete the task will be recorded.

*4-Stair Climb.* Participants will be asked to climb up and down 4 stairs as quickly as possible, using steps with a six-inch rise and ten-inch run. Quality grades will be captured for this test to record the use of railings and manner of ascent (i.e., step-to or step-over-step pattern). The time it takes to complete the task will be recorded.

*Timed Up and Go*. Individuals are asked to rise from a chair, walk 3 meters, turn around, return to the chair, and sit. The time that it takes to complete the task will be recorded. Assistive devices, orthoses, and ankle braces will be allowed.

*Timed Supine to Sit*. Functional axial strength will be assessed by instructing individuals to move from supine to sitting on the edge of a plinth as quickly as possible. The time that it takes to complete the task will be recorded, as well as the manner of sitting (e.g., using one or two hands to pull up).

*Step Test*: This procedure is a test of dynamic standing balance. The participant will maintain stationary balance on one leg while the other foot steps rapidly on and off a 7.5 cm step. The score is the number of repetitions completed in 10 seconds. This test is designed to capture a larger dynamic range of disease progression in participants who are less severely affected, as it is less likely to have ceiling effects.

- - 1. *Measures of strength*

*Quantitative Myometry.* Quantitative Myometry Testing (QMT) will be performed using a force transducer attached to an inelastic strap as previously described. Standardized positions for isometric force testing will be used on five bilateral muscle groups: elbow flexors and extensors, knee flexors and extensors and ankle dorsiflexors.^3^ These muscles were selected for showing good test-retest reliability in our previous natural history studies.

*Manual Muscle Testing (MMT).* Manual muscle testing will be performed on 13 bilateral muscle groups (shoulder abductors, elbow flexors and extensors, wrist flexors and extensors, thumb flexors, hip flexors, extensors, and abductors, knee flexors and extensors, ankle dorsiflexors, and plantar flexors, plus neck extensor and neck flexors). Each muscle group will be scored using a modified MRC scale, in accordance with a protocol that standardizes the subject’s position, direction of movement, and joint angle for resistance, and the examiners stabilization and hand placement.

*Iowa Oral Performance Instrument (IOPI).* The IOPI consists of a pressure bulb attached to a manometer. The pressure bulb will be placed on anterior portion of the tongue and subjects are asked to push the bulb to the hard palate on the alveolar ridge just behind the upper central incisors for 3-5 seconds (repeated 3 times with a 30 second interval between trials). The average of the maximum pressure on three trials will be recorded. Buccal strength will also be quantified bilaterally with pressure bulb placed just inside the cheek. The subject will be asked to purse his/her lips to apply pressure to pressure bulb for 3-5 seconds for a total of 3 separate measures with 30 second rest period. The pressure bulb is single use and disposed of following the three trials.

*Spirometry.* Spirometry is performed in the sitting and supine position, to determine FVC, FEV1, and peak cough flow.

- 1. Patient Reported Outcome Measures

*Myotonic Dystrophy Health Index (MDHI*). The MDHI is a standardized questionnaire to quantify DM1-related symptoms and disease severity.

*Eating Assessment Tool (EAT-10).* The EAT-10 is a 10-item questionnaire designed to assess difficulties with swallowing or dysphagia.

*Domain Delta Questionnaire*. This questionnaire will evaluate the global rate of change, as well as changes in specific domains. Participants will be asked to report whether their health has: 1) Gotten a lot worse; 2) Gotten a little worse; 3) Stayed the same; 4) Gotten a little better; 5) Gotten a lot better. The average change in relevant outcomes will be compared among these five groups. This information will be used as an anchoring procedure to evaluate responsiveness and determine minimally important clinical difference. This questionnaire will be administered at the 12 and 24 month visits. It will also be administered at the 3 month study visit for participants in the biopsy sub-study.

*Work Productivity and Activity Impairment Questionnaire: Specific Health Problem V2.0 (WPAI:SHP).* This brief questionnaire will evaluate the impact of DM1 on a person’s ability to work.

*EQ-5D-SF*: This brief questionnaire will assess over health and impairment.

*Walking Scale-12*: This brief questionnaire will assess walking ability.

*DM1-Activ*: This questionnaire will assess impact of DM1 on daily life.

- 1. Cognitive function/CogState

This protocol will utilize a series of tests from the CogState, a computer-assisted method of cognitive assessment. The specific tests were selected to probe executive dysfunction that occurs in some DM1 patients, based on prior studies and input from neuropsychologists.

*Set-Shifting Test.* This test is a measure of executive function and cognitive flexibility. The participant is presented with a playing card and they have to guess if the card is a “target” card along two dimensions (color or a number). The software provides feedback to the participant and will not display a new stimulus until a correct response is given. The “target” stimulus dimension changes to either an intra-dimensional shift or to a different dimension (extra dimensional shift), and the participant is not told when the set-shifts will occur, and they are continuously required to relearn the target rule to proceed through the test. The multiple set-shifts within this test are pseudo-randomized to create alternate forms of the test. Participants are encouraged to work as quickly and as accurately as possible. The total number of errors is measured across five rounds; a lower score equals better performance.

*Detection Test.* The Detection Test uses a simple reaction time paradigm to measure processing speed. Participants are presented with a playing card on a screen and asked to respond “Yes” by pressing a button as soon as the card flips over to face up. Once a response has been made, the card goes to the back of the pack and a new card is presented. There is a learning period that involves a limited number of correct responses. The participant starts the actual test once the practice is completed. Participants are encouraged to work as quickly and as accurately as possible. Performance speed is measured. Lower scores equal better performance.

*One Back Test.* This is a test of working memory that uses an n-back paradigm. A playing card is presented to the participant facing up in the center of a screen. The participant must decide if the presenting card is identical to the previously presented card. Participants are asked to respond “Yes” or “No” accordingly. There is a practice period followed by the actual test. Participants are encouraged to work as quickly and as accurately as possible. Speed of performance is measured and reaction times are transformed for correct responses. A lower score indicates better performance.

*Identification Test.* This test measures attention by utilizing a choice reaction time paradigm. A card is flipped over face up. The participant must decide whether the card is red or not. If red, he/she presses the “Yes” key, and if not, he/she presses the “No” key. Participants are encouraged to press the appropriate key as quickly and as accurately as possible before the card flips over. Performance speed is measured. A lower score indicates better performance.

*Social-Emotional Cognition Test.* For this test, emotional recognition is measured using an odd-man–out paradigm. Participants are presented with a number of pictures on screen. One of the pictures is different in some way, and the participant must decide which image is different and tap it. Participants are encouraged to work as quickly and as accurately as possible. Accuracy of performance is measured. A higher score indicates better performance.

- 1. Biomarkers and muscle biopsy substudy

*Blood sample*. A blood sample will be collected at each visit to obtain plasma samples for biomarker discovery. The sample may be used to assess plasma RNA, metabolites, or proteins.

Blood tubes to be collected:

- *Yellow top* (8-10 ml) for DNA at baseline, 12 month, and 24 month study visits.
- *PAXgene tube* (2 tubes, 2.5 ml each tube, total 5 ml) for blood RNA at all three visits
- *Yellow top #2* (8-10 ml) for plasma at all three visits

Sample Processing:

*Yellow top* *for DNA.* The preferred method is that samples are placed in a -80 C freezer within two hours of collection. Samples may then be batch shipped to the University of Utah on dry ice. Alternatively, samples may be stored at 4 C for up to two days and then shipped overnight to the University of Utah.

*PAXgene tube*. Once collected, samples need to remain at room temperature for 2 hours. After this, samples may be stored in a -80 C freezer and then batch shipped to the University of Utah on dry ice.

*Yellow top #2*. Samples should be centrifuged for 10 minutes at 4000 RPM at 4 C immediately after collection. The supernatant (plasma) should be aspirated, divided into 3 aliquots, and stored in new tubes. The samples should be placed at -80 C immediately, then batch shipped to the University of Utah on dry ice.

*Urine sample.* The urine sample will be divided in two 5 ml tubes and stored at -80 C for batch shipping.

*Electrocardiogram (ECG).* This will be performed at each visit to evaluate the extent of cardiac conduction disease. PR interval and QRS duration will be determined using standard methods.

*Muscle biopsy sub-study*. The biopsy sub-study will examine RNA splicing defects that are characteristic of DM1. The proposed study builds on results from the initial DMCRN study, in which we obtained paired needle biopsy samples of tibialis anterior muscle from 97 patients. The samples were obtained 3 months apart to approximate the expected time course of muscle sampling in early-phase clinical trials. No significant safety issues were encountered. The biopsy procedure was well tolerated and > 95% of subjects who underwent the initial biopsy returned for the second biopsy. The second biopsy was obtained from the same leg, except in 19 patients whose second biopsy was obtained from the opposite leg. RNAseq of biopsy samples has now been completed and the preliminary analyses indicate that test-retest reliability of splicing biomarkers was substantially better for paired samples obtained from opposite legs. We seek now to confirm this finding in an independent cohort. Furthermore, in discussions with FDA officials about splicing biomarkers, the question was raised whether RNA splicing defects observed in tibialis anterior are representative of other muscles. Therefore, we are also testing whether splicing misregulation in an arm muscle, biceps brachii, is similar to that in tibialis anterior.

*Needle biopsy procedure.* 60 participants will undergo a muscle biopsy at the baseline visit and again at a three month study visit. The biopsy procedure is the same as our previous studies. After skin preparation using povidone-iodine or equivalent antiseptic, the skin and subcutaneous tissues will be anesthetized with 7-10 ml of 0.5% or 1% lidocaine, without epinephrine. The biopsy will be performed using side-cut biopsy needles, core biopsy needles, or the newer Vacora vacuum-assisted biopsy system. For side-cut needles (known as Bergstrom or UCHL needles) or Vacora 14 g biopsy probes, a 3‑mm incision will be made in the skin and muscle fascia, and muscle tissue will be obtained with 1 to 3 passes of the biopsy needle. Closure of the incision is obtained with steristrips or topical skin adhesive. For core biopsy needles (14 gauge, 10 mm length) a skin incision is not required, and 2 to 4 passes of the biopsy needle will be performed. Biopsy specimens (5 to 40 mg) will be frozen in liquid nitrogen within 2 minutes of removal, stored at -70°C, and shipped in batches to the University of Rochester for targeted high-throughput RNA sequencing. A repeat biopsy will be obtained three months later from the opposite tibialis anterior (*n* = 40) or biceps (either side, *n* = 20). The site investigator will determine which site is used for the second biopsy, based on degree of muscle weakness and muscle atrophy. To enable comparisons across muscles having similar weakness, half of the biceps biopsies will be obtained from subjects having at least slight weakness of elbow flexors (MRC score ≤ 4+). Ultrasound may be used to guide needle biopsies of biceps, generally this is not necessary for tibialis anterior.

- 1. Genetic test for CTG expansion at the DM1 locus

Genomic DNA extracted at baseline will be analyzed by PCR, Southern blot, or both, to determine length of the CTG repeat as previously described.^4^ Repeat length may also be determined using higher resolution procedures as they become available. Repeat-primed PCR will be used to test for variant repeats (CCG or CGG repeats embedded within the CTG repeat tract) as previously described.^5^ DNA samples from the 12 and 24 month study visits may be used to assess test-retest reliability of repeat length measurements.

- 1. Genotyping procedure for GWAS

High-density genotyping of common polymorphism (minor allele frequency > 1%) will be performed using Illumina BeadChip microarrays. Genotypes will be used to control for ancestry and relatedness in the cohort, and quality-controlled genotypes will be formatted for association analyses with phenotypes collected.

- 1. Option for study staff to have access to genetic data for future study recruitment.

Genetic confirmation of DM1 is not required for entry in the study. The clinical diagnosis of DM1 is > 99% accurate. Since CTG repeat number does not have an established prognostic value, the current standard of care does not require genetic testing. Accordingly, most DM1-affected individuals in the U.S. have not had genetic testing. For example, 50% of patients in the National Registry of Myotonic Dystrophy (*n* = 947) have not had genetic confirmation. This creates a potential barrier for participation in research studies.

Genetic testing for CTG expansion at the DM1 locus will be performed on all participants in the study. The testing will be performed in a research laboratory, not a CLIA-certified genetic testing laboratory. The test results will not be provided to participants.

Participants in the study will have the option for *DMPK* genetic test results to be placed in the local study binder for future access by site investigators and study staff. For participants who make this selection, the study site staff have the option to reference DM1 genetic test results for future study recruitment. Participants will indicate their selection by checking a box on the consent form. Note that study binders are the only place where DNA test results and personal identifiers are linked. Contact with participants for future studies is exclusively done by sites, and cannot be performed from the central Coordinating Center, which will never have access to patient identifiers (see below). Our goal in providing this option is to eliminate genetic diagnosis as a barrier to participation in research studies.

All participants in the study will have a clinical or genetic diagnosis of DM1. Accordingly, the results of DM1 genetic testing are not expected to impact patient care. In the unlikely event that CTG expansion is not confirmed, the participant may instead have myotonic dystrophy type 2 (caused by repeat expansion in a different gene) or a type of myotonic dystrophy that is not yet described. If this occurs, the subject will be notified and offered arrangements for further neuromuscular evaluation and genetic counseling.

- 1. BioBank of muscle tissue and blood.

Participants in the study will provide their consent for muscle tissue, blood, and urine to be placed in a BioBank. Materials in the BioBank may be used for future unspecified research by investigators within or outside of the Myotonic Dystrophy Clinical Research Network. Materials in the BioBank will include muscle tissue, blood, and urine, and DNA, RNA, or protein derived from these samples. The creation of the BioBank does not imply a commitment to fulfill all requests for research materials, because the quantities of tissue and RNA from needle biopsy samples are limited. BioBank samples will be stored in Drs. Thornton or Johnson’s laboratories in barcoded tubes. When samples are provided to investigators, they can be accompanied by de-identified study data, including age, gender, and indices of disease severity (e.g., muscle strength, timed functional test scores, myotonia scores, MDHI scores). There is no mechanism for laboratory investigators to trace a sample back to individual study participants. The entire sample will be allocated to the BioBank. The first priority for sample analysis is the analysis described in this protocol. Samples that remain can be used for future unspecified research, including proteomic or transcriptomic analyses. Samples distributed to other scientists will not be returned.

- 1. **Safety Assessments**

Assessments that will be performed to evaluate safety are: medical history, physical exam, vital signs, and EKG.

- 1. **Data & Specimen Banking for Future Research Use**

By participating in this study the patient agrees that remaining samples of their muscle tissue, blood, and urine may be kept indefinitely in the Myotonic Dystrophy BioBank at the University of Rochester and University of Utah. The samples may include muscle tissue, blood, urine, or materials derived from these samples, such as protein, RNA, DNA, and cells. The samples will be identified by a code number; not by the patient’s name or other identifying information. The details about future research cannot be described at this time because they depend on scientific advances in the field. The future research may be carried out by qualified scientists in the Myotonic Dystrophy Clinical Research Network or at other facilities. Future researchers who use BioBank samples may be provided with de-identified information about how the patient is affected by DM1, such as muscle strength or walking speed. Researchers will not be provided with information that can directly identify you. Researchers using the samples will be asked to acknowledge the BioBank when they announce results of their studies. However, the patient will not be able to determine whether their samples were used in a particular study, and the patient will not be notified when their samples are provided to researchers. The patient will not benefit financially from potential new discoveries or technological advances brought about through research using samples from the BioBank. The study staff may decide to stop storing materials in the BioBank without sending the patient notice or obtaining their permission.

As part of the BioBank the patient also has the option to allow a cell line to be made from their blood to provide a renewable supply of DNA and other cell components for research. A cell line is a frozen sample of specially processed cells from the patient’s blood that allows researchers to grow more cells and obtain more DNA and other cell components, such as RNA or proteins, for future research. The cell line would not be labeled with the patient’s name, address, birthdate, or other personal identifiers. It is possible that the patient’s cell line would be used in the future by the University of Rochester or research partners for testing or developing a treatment or commercial product. There are no plans to provide financial compensation to the patient should this occur. Patients have the option to check the box in the consent giving permission for have a cell line made from their blood for future research.

- 1. **Genetic/Genomic Research Activities**

Part of the patient’s blood sample will be used for genetic testing. If the patient does not understand what a genetic test is and wishes to receive genetic counseling prior to signing this consent form, they can let the investigator or coordinator know.

The patient’s blood sample will be labeled with an identification code number. The coded sample will be sent to researchers at the University of Utah for isolation of DNA, the genetic material. The patient’s name, address, birth date, and other personal information will not be included with the sample. The researchers at University of Utah or at another site will test the DNA for the gene abnormality that causes DM1. The test will be performed in a research laboratory, not in a certified medical testing laboratory, therefore the results will not be provided to the patient. The patient will have the option for study staff at the University of Rochester to keep their DM1 genetic test results on file for up to eight years. This may eliminate the need for repeat genetic testing in future studies, and the results may be used to identify people who are eligible to take part in future studies. The patient has the option to check the box in the consent form giving study staff at the University of Rochester permission to store their DM1 genetic test result and contact the patient about participation in future studies.

Another part of the patient’s DNA sample will be used by researchers at the University of Utah or another site to test for DNA variants (differences) that are known to occur in human genes. The researchers will be searching for variants that determine how people are affected by DM1. This is an important question because the effects of DM1 are very different from one person to the next. The researchers will receive de-identified information about how the patient is affected, such as their muscle strength, walking speed, and questionnaires. This information will not include the patient’s name, birthdate, address, or other personal identifiers. The information will be matched to the code number of their DNA sample. If researchers are successful in discovering gene variants that affect DM1, the study staff at the University of Rochester may contact the patient in the future to ask about participation in additional studies. Patients have the option to check the box in the consent form giving study staff at the University of Rochester permission to contact the patient about participation in future studies to learn more about DNA (genetic) variants that affect DM1.

- 1. **Costs to the Subject**

There is no cost to research patients to participate in this research study.

- 1. **Payment for Participation**

Patients will be provided an honorarium of $150 for each of the needle muscle biopsies, for a total of $300.

- 1. **Return of Individual Research Results**

The genetic testing will be performed in a research laboratory, not in a certified medical testing laboratory, therefore the results will not be provided to the patient.

1. **CONCOMITANT AND DISALLOWED MEDICATIONS**

Not applicable

1. **SUBJECT WITHDRAWALS**

Possible reasons for withdrawal may include loss of funding, completion of the study, if the patient misses their study visits, or a concern by study staff that participation may have a harmful effect on the patient’s emotional or physical health or welfare.

1. **SAFETY AND REPORTABLE EVENTS**
   1. **Definition of an Adverse Event and Medical Event**

For this study, an *adverse event* is any new, undesirable medical occurrence or change of an existing condition in a subject that occurs during a study visit. An adverse event could be a fall during a 6 minute walk test, a complication during a needle muscle biopsy, etc. We will not capture changes that are occurring as part of the disease progression as an adverse event. These changes will be captured under the Medical History CRF (06) and updated at each visit.

For this study, a *medical event* is any change in symptom, change in frequency, new diagnosis, sickness, hospitalization, surgeries, etc. that happen within the 12 month period that the patient is enrolled in the study. These items will be documented on the Medical History Form (06).

- 1. **Adverse Event and Medical Event Reporting Period**

The recording of adverse events will begin with the start of protocol-specific procedures. Reporting will continue until the last in-person study visit or when a subject ends participation in the study.

- 1. **Reviewing Adverse Events and Medical Events**

Inquiries about medical events will occur at every visit. In addition, subjects will be instructed to contact the study coordinator about any concerns or changes in health status.

Adverse events will be reviewed by with the site investigator and study coordinator at each study visit.

- 1. **Adverse Event and Medical Event Coding**

Coordinators will list a complete description of the adverse/medical event including area of body affected and symptoms. The NMD-CC will code adverse events at the time of data analysis.

- 1. **Adverse Event Follow-up**

The site investigator will review all ongoing adverse events with the subject during each visit and telephone call to evaluate any change in status. All adverse events will be monitored by the treating neurologist until resolution or the end of the study’s AE reporting period. Resolution of an adverse event is defined as a return to the subject’s baseline condition (level of symptoms, laboratory value or frequency of episodes).

The progress of each SAE will be followed by the study PI until resolution or until an appropriate endpoint is reached. An appropriate end point is the resolution of the event or the determination that the event has become chronic or stabilized.

- 1. **Required Reporting Elements**

Details regarding adverse events will initially be entered in the subject’s progress notes as source documentation. Each adverse event will be recorded on the subject’s Adverse Event Log (CRF 56). All of the required data elements included on the Adverse Event Log must be completed for each event. A description of each item required on the Adverse Event Log is described in sections 2.1a – 2.1h.

8.6.a **Adverse Event Description**

Capture a complete description of the adverse event including the area of the body affected as well as symptoms experienced by the patient.

8.6.b **Record Date**

The date an event is recorded is the day that study staff becomes aware of the adverse event either during a study visit, phone call or through the receipt of test results. A complete date must be entered for this field on the Adverse Event Log.

8.6.c **Severity**

The site investigator or study coordinator will grade the severity of each adverse event using the detailed clinical descriptions provided below. This grade will be entered in the appropriate field on the Adverse Event Log.

The general guidelines for grading each adverse event are based on the following scale:

| **Grade** | **Description** |
| --- | --- |
| 1 | Mild; Asymptomatic or mild symptoms; clinical or diagnostic observations only; intervention not indicated. |
| 2 | Moderate; minimal, local or noninvasive intervention indicated; limiting age-appropriate instrumental ADL |
| 3 | Severe or medically significant but not immediately life-threatening; hospitalization or prolongation of hospitalization indicated; disabling; limiting self care ADL |
| 4 | Life-threatening consequences; urgent intervention indicated. |
| 5 | Death related to AE. |

A semi-colon reads as the word ‘or’ within the description of the adverse event’s grade.

8.6.d **Action Taken**

The actions taken to manage adverse events must be recorded using the terms listed in the table below. A minimum of one term must be entered for each adverse event. Multiple actions can be entered.

- None
- Hospitalization
- Terminated from the study
- Unknown
- Medication
  Other

Any changes in concomitant medication must be recorded on the Concomitant Medication Log (CRF26).

The term “Hospitalization” refers only to inpatient hospitalization. “Hospitalization” should not be recorded if a subject was admitted as an outpatient for a procedure. Outpatient procedures can be documented as a response to an adverse event using the code “Other.” The subject’s source documentation will capture information about the procedure.

8.6.e **Seriousness**

The site investigator will evaluate if the adverse event meets the criteria for being a Serious Adverse Event. A Serious Adverse Event (SAE) is defined as any adverse event that:

- Results in death
- Is life-threatening or places the participant at immediate risk of death from the event
- Requires inpatient hospitalization or prolongation of existing hospitalization. Hospitalizations scheduled before enrollment for an elective procedure or treatment of a pre-existing condition which has not worsened during participation in the study will not be considered a serious adverse event.
- Causes persistent or significant disability/incapacity (substantial disruption of one's ability to conduct normal life functions) that is not related to the underlying myotonic dystrophy
- Results in a congenital anomaly/birth defect
- Is a medically important event that required medical intervention to avoid one of the above outcomes

If an AE meets the criteria for being serious, the AE must be listed on the primary Adverse Event Log (CRF56) and additional information must be submitted using the Serious Adverse Event Form (CRF57). Additional instructions on reporting Serious Adverse Events are located in section 3.0.

8.6.f **Outcome**

Resolution of an adverse event is defined as a return to the subject’s baseline condition (level of symptoms, laboratory value or frequency of episodes). Otherwise, mark the outcome as “ongoing.”

8.6.g **Start Date**

The start date is the day an adverse event begins

If study personnel are unable to determine an exact date based on the subject’s report or available records, the study personnel must estimate a start date using the information available. Additional details for entering dates are included in the study manual section, Data Collection and Documentation.

8.6.h **End Date**

The end date is the day an adverse event resolves or the subject returns to their baseline condition.

If study personnel are unable to determine an exact date based on the subject’s report or available records, the study personnel must estimate an end date using the information available.

At the end of the study, any adverse events that have not resolved will be recorded as “ongoing” on the Adverse Event Log (CRF56).

An end date will only be entered for adverse events that have resolved. Resolution of an adverse event is defined as a return to the subject’s baseline condition.

8.6.i **Neurologist Review**

The site investigator will review the Adverse Event Log (CRF56) after each visit to ensure that all adverse events have been entered correctly.

**8.7 Medical Event Coding Guidelines**

8.7.a **Preexisting conditions**

All preexisting conditions and prior medical history must be documented on the Medical History Form (CRF06).

8.7.b **Changes in Severity or Frequency**

All medical events that increase in severity or frequency during the reporting period must be reported in the Medical History Form (06). The start date for the new medical event will be the date the change in severity occurred.

8.7.c **Diagnostic Procedures and Surgery**

Diagnostic procedures or surgery necessitated by an underlying medical condition should not be reported as adverse events. This information will be captured in the Medical History Form (06). The underlying medical condition which made these procedures necessary should also be recorded on the Medical History Form (06).

8.7.d **Terminology for Medical Events**

The medical event term used for conditions that present with multiple different symptoms should be the diagnosis, not each individual symptom. For example, an Upper Respiratory Infection should be recorded rather than each individual symptom for a cough, sore throat and fever.

**8.8 Serious Adverse Events**

8.8.a **Serious Adverse Event Definition**

A Serious Adverse Event (SAE) is defined as any adverse event that:

- Causes persistent or significant disability/incapacity (substantial disruption of one's ability to conduct normal life functions) that is not related to the underlying myotonic dystrophy
- Results in death Is life-threatening or places the participant at immediate risk of death from the event Requires inpatient hospitalization or prolongation of existing hospitalization. Hospitalizations scheduled before enrollment for an elective procedure or treatment of a pre-existing condition which has not worsened during participation in the study will not be considered a serious event.
- Results in a congenital anomaly/birth defect
- Is a medically important event that required medical intervention to avoid one of the above outcomes

**8.9 Serious Adverse Event Notification Process**

1. The site investigator or study coordinator must contact the study PI and the NMD DMCRN Project Coordinator within 24 hours of their first knowledge of the event by email.

|  | **Study Principal Investigator (PI)** | **Project Coordinator** |
| --- | --- | --- |
|  | Charles Thornton | Jeanne Dekdebrun |
| Email | Charles_Thornton@  urmc.rochester.edu | Jeanne_Dekdebrun@  urmc.rochester.edu |
| Fax | 585-273-1255 | 585-273-1255 |
| Phone | 585-275-2542 | 585-276-4611 |

2. At the time of the initial notification of the SAE or within 24 hours, the site must submit a copy of the Serious Adverse Event Form (CRF57) to the PI and DMCRN Project coordinator. The forms can be sent as a PDF email attachment or by fax. The NMD-CC SAE Notification fax cover sheet should be used when faxing these documents. This form is included in the study manual appendix.

**8.10 Required Reporting Elements for an SAE Submission**

The following details must be included on the Serious Adverse Event Form (CRF57):

- Subject identifiers including the subject’s study ID number and initials
- A diagnosis or if a diagnosis is unknown, the individual symptoms can be listed in the initial report
- The name and contact information for the site investigator that is reporting the SAE to the study sponsor

**8.11 Study PI SAE Review**

All SAEs will be reviewed by the study PI, who will complete the Serious Adverse Event Review Form (CRF59).

**8.12 Notifying Regulatory Authorities**

Site coordinators will report SAEs to their IRB as outlined by site IRB guidelines.

**8.13 SAE Follow-up Reports**

The site investigator must provide any updated information about the SAE during the reporting period until the event has resolved or stabilizes. Any new pertinent information on an SAE obtained after the initial submission of the SAE Form (CRF 57) will be recorded on the SAE Follow up Form (CRF 58).

1. The SAE Follow-up Form (CRF58) will be completed by the site investigator or study coordinator. The site will include deidentified copies of any new relevant medical records such as a discharge summary, hospital notes and laboratory results.

2. The site will send a copy of the SAE Follow-up Form (CRF58) and other documentation to the PI and NMD-CC project coordinator. The documents can be sent as a fax or emailed as a PDF attachment. The NMD-CC SAE Follow-up fax cover sheet should be used when sending these documents. This form is included in the Appendix of the Study Manual.

3. The study PI will review the information that was submitted. Additional SAE Review Forms (CRF59) will be completed by the study PI if:

- - - - The classification of the event changes (relatedness or expectedness)
      - The event has resolved or an appropriate endpoint has been reach (resolution or determination of the event to be chronic and/or stabilized)

**8.14 SAE Data Management**

Original copies of all CRFs generated for the SAE must be entered in REDCap.

The information that is reported on Serious Adverse Event Form (CRF57) and SAE Follow up Form (CRF58) must correspond to the information that is entered on the subject’s primary Adverse Event Log (CRF56). The adverse event term, dates and ratings must be consistent between forms. It is the site’s responsibility to ensure that all data is reported accurately. The data manager will review the data that was submitted and will query any fields where there are discrepancies.

If a discrepancy is noted by study personnel, the error will be corrected in the database. If a discrepancy is discovered by the NMD-CC, the site will be sent a Data Clarification Form.

1. **RISK/BENEFIT ASSESSMENT**
   1. **Potential Risks**

The procedures described pose minimal risk. Release of medical information prior to participation could result in a potential loss of privacy.

There is a small risk of bruising from blood draws and muscle soreness from the QMT, MMT, maximum voluntary isometric contraction grip testing, and functional tests.

Risks of the muscle biopsy include bruising, pain, and muscle soreness in the area of the biopsy. There may be a small, permanent scar on the skin from the incision. There will also be the risk of bleeding, infection, and numbness (from touching a nerve with the needle). There is the rare risk that a person might be allergic to lidocaine with reactions that could range from mild skin rash to anaphylactic shock (drop in blood pressure, breathing difficulties).

- 1. **Protection Against Risks**

All patients will be monitored closely by the study site nursing staff and Investigators during the procedures to prevent and/or minimize any potential risks or discomfort. The nursing staff and study personnel will receive in-service training regarding these procedures. Each participant will be asked to report any continuing discomfort caused by any of the procedures.

Patients will be withdrawn from the study if intolerable side effects or serious AEs, as determined by the Investigator, are identified. Patients who cannot tolerate the procedures or who develop other illnesses that affect the evaluations may be withdrawn from the study. Patients may withdraw at any time.

- 1. **Potential Benefits to Subjects**

There are no direct benefits that patients may expect to receive as a result of participation in this study.

- 1. **Alternatives to Participation**

There are no alternatives to participation.

1. **CONFIDENTIALIATY OF DATA AND INFORMATION STORAGE**

The underlying source documents (consent, medical records, and case report forms) for the study are maintained in the study binder for each subject at each site. Study binders are kept in secured areas, accessible only to authorized study personnel.

The study sites and their partner(s) and designee(s), ethics committees, and various government health agencies, including representatives of Biogen Idec, may inspect the records of this study. De-identified data from the study will be shared with the partners of this study for the purpose of planning future clinical studies and trials. Scientists at Biogen Idec will have potential to link deidentified study data with serum, plasma, RNA, and cDNA samples that they receive. Every effort will be made to keep the patient’s personal medical data confidential.

1. **RESEARCH INFORMATION IN MEDICAL RECORDS**

Indicate if any research data will be included in the subject’s medical record (e.g., lab test results, drug assignment, or indication of study participation)

1. **DATA ANALYSIS AND MONITORING**
   1. **Sample Size Determination**

Our sample size is designed to capture a clinically meaningful change in our key endpoints: 30-foot go, grip strength, ADF strength, and supine FVC. In order to estimate a sample size based on the continuous variable of change in time in the 30-foot go, the following assumptions were made to capture precision: a mean change of 0.177 with a standard deviation of 1.00. With an alpha of 0.05 and a two-sided 95% confidence interval, a total sample size of 500 subjects is needed to compare two subgroups of 225 individuals each in the mild DM1 and the more severely affected DM1 groups. This sample size accounts for a 10% drop out rate, as was identified in the prior DMCRN study. This sample size would also allow for computation of sensitivity and specificity of the primary endpoints to discriminate these two subgroups of individuals.

- 1. **Planned Statistical Analysis**

This analytical plan is for primary and secondary objectives. The variability of biomarkers and we will evaluate our endpoints for responsiveness to change over time. We will use the mean and standard deviations to construct 95% confidence intervals for each visit. We will evaluate the significance of any change over 12 and 24 months. For responsiveness we will look at the effect size and the standardized response mean.^6^ The effect size is defined as the average of the difference between end-of-treatment and baseline scores divided by the SD of the baseline scores. The standardized response mean is defined as the mean change divided by the standard deviation of the changes from baseline. For within-group (paired) comparisons, as is the case here, the standardized response mean is equivalent to the paired t-test (the two differ only by a factor of the square root of the sample size). We will compare responsiveness between all outcome measures.

Both anchor-based and distributional methods for determining the minimal clinically important difference (i.e., the minimum degree of change in scores that are deemed significant) will be used. The standard error of the measurement (SEM) will be calculated by multiplying the SD of the baseline measurement by the square root of the difference of 1 minus the ICC of the measure.^7^ The 90% minimal detectible change is calculated by multiplying the standard error of the measurement by the the square root of 2 by the Z-score for a cut-off of 90% distribution (1.65). A multiple anchor technique will also be utilized to describe the responsiveness of the motion analysis metric as described by Guyatt et al. and elsewhere.^8-10^ The clinical outcomes will be compared among the categories of “domain delta” (e.g.: unchanged, a little better, a lot better, etc.) using the Jonckheere-Terpstra test. This test, which is designed to detect differences among ordered classes, is more powerful than tests that do not account for the ordering among the classes, such as the Kruskal-Wallis test. This method can also be used to help determine the minimal important difference (MID). The responsiveness to change index will be assessed by dividing the absolute change scores in the DM1 group with stratification at the subgroup level (e.g., age, gender). All statistical significance will be set at alpha of 0.05 at two-tailed. Go-no go decision point for each outcome measure will be set for a responsive index of at least 0.5. This decision will be reviewed by the study investigators and advisory panel periodically during the project period and adjusted as necessary.

- 1. **Data and Safety Monitoring**

Due to the routine risks associated with this protocol and lack of therapeutic interventions, the proposed study will not require a Data Safety and Monitoring Board. The Coordinating Investigators (Drs Thornton and Johnson) will be responsible for the safety monitoring of the study. Summary data regarding complications from the muscle biopsy procedure and results of post-biopsy follow up calls will be tabulated and reviewed every 3 months.

- - 1. Data management

Data management for the study will utilize Research Electronic Data Capture (REDCap), a software toolset and workflow methodology for electronic data collection. REDCap was developed at Vanderbilt University in conjunction with a Consortium of international partners, including CTSI sites throughout the United States. REDCap is a secure, web-based application that provides an intuitive interface for entering study data. Data entry is subject to real-time validation rules (e.g., automated recognition of data type, range checks). REDCap provides management tools and audit trails for reporting, monitoring and querying of study records, as well as capability for automated export to common statistical packages (SPSS, SAS, Stata, R/S-Plus). Through its partners in the Consortium, Vanderbilt has disseminated REDCap to 734 academic and non-profit institutions on six continents with over 100,000 research end-users (www.project-redcap.org). The CTSI Informatics Core at the University of Rochester, a unit of the Academic Information Technology (AIT) Group, actively supports REDCap. REDCap servers are housed in a secure data center at the University of Rochester. The AIT Group is responsible for the security of the network server, including firewalls, virus checking, access passwords, backup, and disaster recovery. All web-based data transmission for REDCap is encrypted. REDCap was developed in a manner consistent with HIPAA security requirements. In addition to these security layers, each study site requires individual application passwords for study staff. Access to study data is restricted to staff members with a direct need.

To prevent loss of privacy data, information obtained in the study will be stored on file servers maintained by the University of Rochester Information Systems Division. This group is responsible for the security of all University network servers, including firewalls, virus checking, network and workstation access passwords, and backup and disaster recovery. In addition to these security layers, the University of Rochester Neuromuscular Disease Center further safeguards data privacy by requiring individual application passwords and restricting access of confidential data to only those staff members with a direct need. Individual identifiers are limited to a single data table. All other files are linked by a non-informative, random identifier.

The underlying source documents (consent, medical records, and case report forms) for the study are maintained in the study binder for each subject at each site. Study binders are kept in secured areas, accessible only to authorized study personnel. Every effort will be made to keep the patient’s personal medical data confidential.

The study sites and their partner(s) and designee(s), ethics committees, and various government health agencies, including the Food and Drug Administration, National Center for Advancing Translational Sciences, and National Institute of Neurological Disorders and Stroke, may inspect the records of this study. De-identified data from the study may be shared with the partners of this study, including the Food and Drug Administration, National Center for Advancing Translational Sciences, and National Institute of Neurological Disorders and Stroke, for the purpose of planning future clinical studies and trials, for interpreting results of the study, and to verify that the study was carried out correctly. De-identified data from the study may be shared with pharmaceutical companies for the purpose of planning clinic trials in DM1.

- - 1. Data sharing between studies

Some participants in the study may have participated in prior natural history studies of the DMCRN or its participating sites. Data from these participants may be shared between studies if the participant selects the appropriate check box on the consent form. Participants will also have the opportunity to be contacted for future studies, if they select the appropriate check box on the consent form. Blood samples will be stored at the University of Utah for future use by investigators, as detailed in section 4. Muscle samples will be stored at the University of Rochester until processed. Data obtained from the study will be available for use by all members of the DMCRN. Summary data will be provided to all members of the DMCRN at the completion of the study. As noted in the biobank section, biobank materials will be available to DMCRN investigators and outside investigators once the Aims have been completed. Data from the study will be made available to the research community through publications. Publications from the study will include summary data and may include individual data for disease severity, such as, strength, timed tests of mobility, or splicing changes. Publications will not include personal identifiers. If published, individual data on age will be scrambled so that age-at-entry is not unique.

1. **REFERENCES**

1. Goldman A, Ramsay M, Jenkins T. Ethnicity and myotonic dystrophy: a possible explanation for its absence in sub-Saharan Africa. Ann Hum Genet 1996; 60(Pt 1):57-65.

2. Griggs RC, Wood DS. Criteria for establishing the validity of genetic recombination in myotonic dystrophy. Neurology 1989;39(3):420–1.

3. Personius KE, Pandya S, King WM, Tawil R, McDermott MP. Facioscapulohumeral dystrophy natural history study: standardization of testing procedures and reliability of measurements. The FSH DY Group. Phys Ther 1994;74(3):253–63.

4. Thornton CA, Johnson K, Moxley RT. Myotonic dystrophy patients have larger CTG expansions in skeletal muscle than in leukocytes. Ann Neurol 1994;35(1):104–7.

5. Braida C, Stefanatos RKA, Adam B, et al. Variant CCG and GGC repeats within the CTG expansion dramatically modify mutational dynamics and likely contribute toward unusual symptoms in some myotonic dystrophy type 1 patients. Hum Mol Genet 2010;19(8):1399–412.

6. Guyatt G, Walter S, Norman G. Measuring change over time: assessing the usefulness of evaluative instruments. J Chronic Dis 1987;40(2):171–8.

7. Wyrwich KW, Tierney WM, Wolinsky FD. Further evidence supporting an SEM-based criterion for identifying meaningful intra-individual changes in health-related quality of life. J Clin Epidemiol

1999;52(9):861–73.

8. Guyatt GH, Osoba D, Wu AW, Wyrwich KW, Norman GR. Methods to explain the clinical significance of health status measures. Mayo Clin Proc 2002;77(4):371–83.

9. Jaeschke R, Singer J, Guyatt GH. Measurement of health status. Ascertaining the minimal clinically important difference. Control Clin Trials 1989;10(4):407–15.

10. Mathai SC, Puhan MA, Lam D, Wise RA. The minimal important difference in the 6-minute walk test for patients with pulmonary arterial hypertension. Am J Respir Crit Care Med 2012;186(5):428–33.

Appendix 1. Order of Testing to be performed by clinical evaluator

1. MMT
2. QMT
3. Grip strength
4. FVC
5. Timed supine to sit
6. 30 foot go
7. Timed Up and Go
8. Ascend 4 stairs
9. Step test
10. 9 HPT
11. Picking up coins
12. vHOT
13. Iowa Oral Performance Instrument
